# Can the adapted arcometer be used to assess the vertebral column in children?

**DOI:** 10.1590/bjpt-rbf.2014.0060

**Published:** 2014

**Authors:** Juliana A. Sedrez, Cláudia T. Candotti, Fernanda S. Medeiros, Mariana T. Marques, Maria I. Z. Rosa, Jefferson F. Loss

**Affiliations:** Escola de Educação Física, Universidade Federal do Rio Grande do Sul (UFRGS), Porto Alegre, RS, Brazil

**Keywords:** physical therapy, evaluation, spine, children, validity of tests

## Abstract

**BACKGROUND:**

The adapted arcometer has been validated for use in adults. However, its suitability for use in children can be questioned given the structural differences present in these populations.

**OBJECTIVE:**

To verify the concurrent validity, repeatability, and intra- and inter-reproducibility of the adapted arcometer for the measurement of the angles of thoracic kyphosis and lumbar lordosis in children.

**METHOD:**

Forty children were evaluated using both sagittal radiography of the spine and the adapted arcometer. The evaluations using the arcometer were carried out by two trained evaluators on two different days. In the statistical treatment, the intraclass correlation coefficient (ICC), Pearson's product moment correlation, Spearman's rho, the paired t test, and Wilcoxon's test were used (α=.05).

**RESULTS:**

A moderate and significant correlation was found between the x-ray and the adapted arcometer regarding thoracic kyphosis, but no correlation was found regarding lumbar lordosis. Repeatability and intra-evaluator reproducibility of the thoracic kyphosis and lumbar lordosis were confirmed, which was not the case of inter-evaluator reproducibility.

**CONCLUSION:**

The adapted arcometer can be used to accompany postural alterations in children made by the same evaluator, while its use for diagnostic purposes and continued evaluation by different evaluators cannot be recommended. Further studies with the aim of adapting this instrument for use in children are recommended.

## Introduction

The early identification of spinal alterations is fundamental, particularly in childhood, because during this phase such alterations are unconsolidated and may therefore be delayed or even reverted^1^. To classify postural alterations and follow up any treatment, an accurate assessment of the spinal curvature is essential, given that treatments are generally based on the degree of curvature and its progression^2^.

Generally, physiotherapeutic postural evaluation employs methods based on observation that do not permit objective quantification of the degree of alteration, which constitutes a limitation in clinical practice. The need for early quantitative identification of postural alterations, without overexposing the patient to radiation, has encouraged the development of non-invasive instruments designed to objectively measure the curvature of the spine and postural alterations^3^^-^ 5.

The choice of assessment instrument should be based on scientific parameters, such as precision, accuracy, concurrent validity, repeatability, reproducibility, and the diagnostic capacity of the measurements provided. In addition, the choice should also consider practical parameters, such as ease of transport and ease of use of the instrument, in order to ensure that the patient can be assessed quickly and comfortably^6^. The arcometer proposed by D'Osualdo et al.^7^ in 1997 for the assessment of the thoracic spine incorporates most of these features. Recently, Chaise et al.^5^ proposed modifications to the structure of the original instrument and to the method used to calculate the spinal curvature and were, thus, also able to validate its use in the lumbar spine^5^. Although the original instrument was assessed in a younger sample^7^, the concurrent validity and intra- and inter-evaluator reproducibility of the adapted arcometer have only been confirmed in an adult population^5^.

However, given the structural differences between adults and children, such as the size of the trunk and the magnitude of the spinal curvature, the applicability of this instrument in this specific population may be questioned. Hence, the objective of this study was to verify the concurrent validity, repeatability, and inter- and intra-evaluator reproducibility of the adapted arcometer when assessing the angles of sagittal curvature in the spines of children.

## Method

The sample consisted of 40 individuals, 15 female and 25 male, average age 10.7±2.7 years, average body mass 38.7±13.1 kg, and average height 1.39±0.17 m. The sample size was calculated using GPower Software with effect size of 0.5, a probability error of 5%, and power test of 95%, resulting in a recommendation of 34 individuals. Six children were added to ensure sufficient sample size during the data collection period. With the child's agreement, the parents signed an informed consent form authorizing participation in the study, which was approved by the Ethics Committee of Universidade Federal do Rio Grande do Sul (UFRGS), Porto Alegre, RS, Brazil, under the number 19685.

The assessment consisted of two procedures: a panoramic X-ray examination of the vertebral column and an evaluation using the adapted arcometer^5^. The X-ray was carried out in the sagittal plane, while the child stood still with the shoulders and elbows flexed at 90 degrees. Based on the X-ray, the angles of the thoracic and lumbar curvatures were calculated using the two-line Cobb method^8^^,^ 9. To obtain the Cobb angle (CA) of the thoracic curvature, the upper vertebral plateau of T1 and the lower vertebral plateau of T12 were marked, and for the CA of the lumbar curvature, the upper vertebral plateau of L1 and the lower vertebral plateau of L5 were marked. Two independent evaluators carried out all of the procedures to obtain the CA for each participant on two different occasions. Based on the assumption in the literature that five degrees is considered the mean error when measuring the CA^10^, in those cases in which the measurements obtained for a particular participant varied by more than five degrees, either between the evaluators or between the measurements obtained by the same evaluator, a new evaluation was performed. The mean values of the angles obtained were used in the statistical analyses.

**Figure 1:**
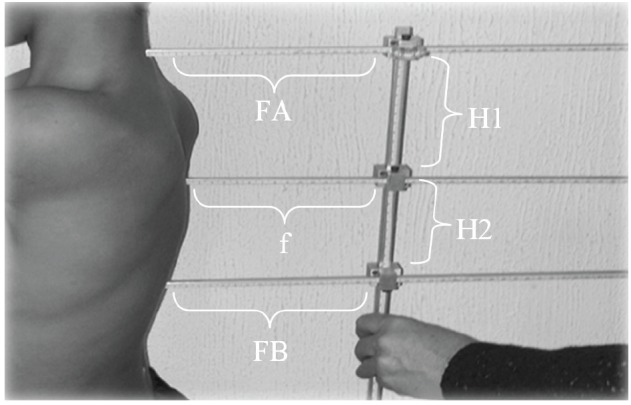
The adapted arcometer being used to measure thoracic kyphosis. H1: distance between T1 spinal process and the apex of the curvature. H2: distance between the apex of the curvature and T12 spinal process. FA, f and FB: upper rod, central rod and lower rod, respectively.

To evaluate the thoracic kyphosis and lumbar lordosis with the adapted arcometer, as with the X-ray examination, the child stood still with the shoulders and elbows flexed at 90 degrees. The spinal process of T1 and T12, and L1 and L5 respectively, were identified by means of palpation. The upper rod (FA) and the lower rod (FB) of the adapted arcometer were positioned on the palpated spinal process and the central rod (f) was positioned on the apex of the curvature. Figure 1 illustrates the position of the adapted arcometer when evaluating thoracic kyphosis. Based on the measurements obtained with the adapted arcometer, the angles of the sagittal curvature of the spine were calculated using trigonometry, according to the method described by Chaise et al.^5^.

**Figure 2:**
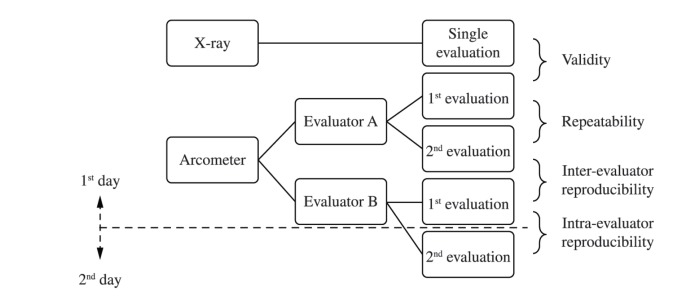
Schematic diagram showing the evaluations conducted using the adapted arcometer and X-rays.

Two trained evaluators (evaluator A and evaluator B) performed the evaluations with the adapted arcometer on two different days, with a minimum interval of one day and maximum interval of ten days. Evaluator A assessed the children twice on the same day (to verify the repeatability) while evaluator B assessed the children twice on two different days (to verify intra-evaluator reproducibility). For the concurrent validity, the Cobb angle results of the thoracic and lumbar spine were used together with the results obtained by evaluator A in the first evaluation, and to verify the inter-evaluator reproducibility, the results from the second evaluation of evaluator A were compared with those obtained by evaluator B in the first evaluation (Figure 2). The statistical treatment was conducted using SPSS version 17 software. The normality of the data was assessed using the Shapiro-Wilk test. The paired t-test or Wilcoxon test was used to verify the differences between measurements. Intraclass Correlation Coefficient (ICC), Pearson's product-moment correlation or Spearman's rho was used to calculate the correlation between measurements. The correlation rates were classified as trivial (.00 to .10), small (.10 to .30), moderate (.30 to .50), large (.50 to .70), very large (.70 to .90), and practically perfect (.90 to 1.00)^11^. The level of significance adopted in all the tests was .05.

## Results

The results of the evaluations for thoracic kyphosis and lumbar lordosis carried out using the adapted arcometer showed no significant difference when compared with the evaluations based on X-rays (Table 1). Regarding the tests of repeatability and intra- and inter-evaluator reproducibility, there were no significant differences in terms of either thoracic kyphosis or lumbar lordosis (Table 1).

**Table 1:** Average values and standard deviations (SD) of the different evaluations made with X-ray and adapted arcometer.

Evaluated aspect	Evaluation	Thoracic kyphosis		Lumbar lordosis	
		Average±SD (°)	p	Average±SD (°)	p
Concurrent validity	X-ray Cobb angle	49.4±11.2	0.131^a^	42.1±8.7	0.070^b^
	Evaluator A 1^st^ evaluation	53.6±11.5		39.7±22.2	
Repeatability	Evaluator A 1^st^ evaluation	53.6±11.5	0.349^a^	39.7±22.2	0.791^b^
	Evaluation A 2^nd^ evaluation	51.8±12.1		39.1±19.8	
Inter-evaluator reproducibility	Evaluation A 2^nd^ evaluation	51.8±12.1	0.640^a^	39.1±19.8	0.361^b^
	Evaluator B 1^st^ evaluation	53.2±8.8		36.3±19.0	
Intra-evaluator reproducibility	Evaluator B 1^st^ evaluation	53.2±8.8	0.643^a^	36.3±19.0	0.762^b^
	Evaluator B 2^nd^ evaluation	53.2±10.1		30.5±18.2	

aPaired t test

bWilcoxon test.

When the correlation between the measurements obtained with the adapted arcometer and those obtained with X-rays were evaluated, there was only a moderate correlation for thoracic kyphosis, while for lumbar lordosis the correlation was not statistically significant. Similarly, the inter-evaluator reproducibility was not statistically significant for either thoracic kyphosis or lumbar lordosis. The correlations between the remaining evaluations can be classified as moderate (Table 2).

**Table 2:** Statistical results referring to the correlations between the different evaluations.

Region	Evaluated aspect	Variable	Correlation test	p
Thoracic kyphosis	Concurrent validity	X-ray vs. Eva A (1^st^)	0.407^a^	0.009*
	Repeatability	Eva A (1^st^) vs. Eva A (2^nd^)	0.439^b^	0.002*
	Inter-evaluator reproducibility	Eva A (2^nd^) vs. Eva B (1^st^)	0.257^b^	0.052
	Intra-evaluator reproducibility	Eva B (1^st^) vs. Eva B (2^nd^)	0.504^b^	0.001*
Lumbar lordosis	Concurrent validity	X-ray vs. Eva A (1^st^)	0.037^c^	0.983
	Repeatability	Eva A (1^st^) vs. Eva A (2^nd^)	0.445^b^	0.002*
	Inter-evaluator reproducibility	Eva A (2^nd^) vs. Eva B (1^st^)	0.258^b^	0.052
	Intra-evaluator reproducibility	Eva B (1^st^) vs. Eva B (2^nd^)	0.433^b^	0.003*

Eva A - evaluator A; Eva B - evaluator B; 1st - first evaluation; 2nd - second evaluation

aPearson's r

bICC

cSpearman's rho

*significant correlation (p<0.05).

## Discussion

The aim of the present study was to verify the validity, repeatability, and intra- and inter-evaluator reproducibility of the adapted arcometer when used to measure the angles of sagittal curvature in the spine of children. To achieve this, the study conducted by Chaise et al.^5^ with adults was used as reference. In that study, the adapted arcometer was found to provide valid and reproducible results in both the intra- and inter-evaluations^5^. By contrast, in the present study, when used to evaluate children, the adapted arcometer did not present good levels of concurrent validity or inter-evaluator reproducibility, which indicates it is inappropriate for use in the diagnosis of postural alterations in the spine of children and for clinical follow-up when performed by different evaluators. Despite this, the instrument presented adequate repeatability and intra-evaluator reproducibility, which indicates that it is appropriate for use in the clinical follow-up conducted by the same evaluator.

Despite the existence of non-invasive methods, when attempting to determine the position of the spine, the X-ray will probably remain the most accurate method and, therefore, the gold standard diagnosis and treatment follow-up method^12^. However, the X-ray depends on advanced technological resources and is often inappropriate for routine use, as the individual is exposed to physical risk^13^. Consequently, a variety of methods has been used to evaluate spinal curvature. This evaluation is equally important for diagnostic purposes, to accompany postural alterations to the spine, and assess the efficacy of treatments. Among the non-invasive instruments and methods used are DIPA (Digital Image-based Postural Assessment), which is a postural evaluation software based on photogrammetry^14^, kypholordometry^15^^,^ 16, Moiré's topography^17^, the flexible ruler^6^^,^ 18 ^,^ 19, the plumbline distance^20^^,^ 21, the Inclimed^21^, and the arcometer^7^.

Two studies in the literature consider the validation aspects of the arcometer. D'Osualdo et al.^7^, the first to describe the method in their evaluation of children with different degrees of kyphosis, obtained excellent correlations for validity (r=.98), intra-evaluator reproducibility (r=.99), and inter-evaluator reproducibility (r=.99) and consequently suggest that the arcometer can be used to accompany postural alterations to the thoracic spine. The second study, by Chaise et al.^5^, proposed structural modifications to the original instrument that provided a greater degree of freedom in upper and lower rods, thus allowing them to present different lengths. The alteration to the length of the rods led to the modification of the method of calculating the angle of the curvature, which could then be carried out considering two distinct arcs. With these modifications, Chaise et al.^5^ improved the original proposal and thus also managed to validate the instrument for use in measuring lumbar curvature. However, the very strong and significant correlation found for the validity of thoracic curvature (r=.94, p<0.01) and the strong and significant correlation found for the validity of lumbar curvature (r=.71, p<0.01) were only verified in an adult sample.

Given that in the present study there is a considerable difference in the age, body mass, and height of the sample in relation to that of Chaise et al.^5^, these characteristics may explain the divergent results obtained between the studies, since the evaluators were previously duly trained in both the palpation technique and the collection protocol with the adapted arcometer. Moreover, the greater variability in terms of body posture and the greater flexibility of the spine in the young, could also partially explain the contrasting results in this and the cited papers with older subjects, since the position used in both exams was the same.

Furthermore, if the estimated error, due to variation in the execution of the protocol (palpation, positioning the rods, etc), is considered the same in adults and children, the repercussion of the error in the calculated angle will be proportionally much greater in children. For example, when measuring an adult, a 1 cm error represents less than 10% of the distance between the rods, while in children the same error could represent more than 40%, due to the size of the trunk. Moreover, when using the arc tangent to calculate angles, the smaller the value using this trigonometric function the greater the impact any error will have on the estimated angle. In adults, the numbers used as input in the arc tangent function will be approximately 1 unit, while in children it will be approximately 0.5. If we have 0.1 of variance in 1 unit (from 1.0 to 1.1), the angle calculated using the arc tangent will change from 45.0° to 47.7°. By contrast, the same variation of 0.1 in 0.5 (from 0.5 to 0.6), the angle calculated using the arc tangent will change from 26.5° to 30.9°. These differences arise from variations in the positions of the rods when placed on the spine. Therefore, due to the variations that occur over short lengths of the trunk, there is a clear need to find a more appropriate procedure that can be used in children. For example, when using the adapted arcometer in clinical practice, the risk of error could be reduced by registering the length of the rods and maintaining the same length during a second evaluation. This issue is particularly important when one considers the intrinsic postural variability of children and adolescents. It should be noted that the results assessed herein refer to a specific range of thoracic and lumbar curvatures. Thus, the fact that this study did not evaluate straighter or more accentuated curvatures may be considered a limitation.

## Conclusion

While the adapted arcometer can be used to quantify the thoracic and lumbar curvatures of adults in the sagittal plane, to date it has not been possible to validate and establish inter-evaluator reproducibility for its use in children, making it unsuitable for diagnostic purposes and in the follow up of postural alterations performed by different evaluators in this population. However, as the adapted arcometer has been shown to have intra-evaluator reproducibility it can be used by the same evaluator in the clinical situation to monitor spinal curvature in children. Nevertheless, further studies designed to adapt this instrument for use in children are necessary.
